# The mental workload of conducting research in assessor cognition

**DOI:** 10.1007/s40037-019-00549-0

**Published:** 2019-11-19

**Authors:** Andrea Gingerich, Peter Yeates

**Affiliations:** 1 Northern Medical Program, University of Northern British Columbia, Prince George, British Columbia, Canada; 2 Medical School Education Research Group (MERG), Keele University School of Medicine, Keele University, Keele, UK; 3 Department of Acute Medicine, Fairfield General Hospital, Pennine Acute Hospitals NHS Trust, Bury, Lancashire, UK

In this issue, Paravattil and Wilby review the literature on assessor mental workload in health professions education [1]. Their review highlights an apparent paradox: theoretically-based interventions that aimed to reduce mental workload have successfully improved markers of assessment quality, but the hypothesized mediating influence of cognitive load has not been demonstrated despite the use of two separate measures in multiple studies. We agree with their interpretation that this points to an issue with the measurement methods or with the theorized model. Or with both. This is the type of quandary that we regularly encounter in assessor cognition research and we could not resist further exploration of it.

Studying cognition during complex tasks is difficult. Since cognition is neither directly measurable, nor sufficiently available to introspection to enable fully accurate self-report, researchers have had to use proxies and make assumptions in the methodological techniques. The studies cited in Paravittal and Wilby’s review used either ‘secondary task’ measures (i.e. how rapidly a participant responds to an unrelated stimulus) or the NASA-TLX, a self-reported measure of mental workload, or a combination of both. Both approaches are well established and have been used extensively in other fields. As discussed, Naismith and Cavalcanti found the validity of these and other measures of cognitive load in a medical education context to be far from ideal [2]. Within cognitive neuroscience, researchers studying mental workload may triangulate several physiological measures with subjective and secondary task measures to gain greater confidence [3], but regardless, measurement of mental workload is difficult and imperfect. As a result, the paradoxical finding highlighted above may emanate from a limitation of measurement for this purpose in this particular context. In other words, cognitive load was impacted by the intervention but the methods were not sufficiently sensitive to measure the change.

Conversely, an alternative explanation for the findings is possible and must be considered. Since cognition cannot be directly observed, researchers use findings to build theoretical models of cognitive processes. The models are useful in several ways including helping us to understand our own thinking and making predictions for how we will likely respond in particular circumstances. Hypotheses based on these models are used to inform research questions and research methods to further specify the original model or to initiate the construction of new models. As such, all models are our latest best guesses, and are at least somewhat wrong, but some are useful [4]. The paradoxical finding that assessment outcomes improve when interventions aimed to decrease mental load are used, even though the interventions have no measurable impact on cognitive load, may emanate from a limitation of the cognitive model of mental workload and cognitive load. For example, in psychology, cognitive load has been understood to exert its influence on a person’s thinking within what is known as ‘dual process’ cognition [5]. This extremely well-established theory models cognitive load as consuming the resources of effortful conscious processes (system 2) so that when a person’s mental workload exceeds a threshold they are unable to deliberately attend to or process further information which impairs their task performance and makes them rely more fully on automatic (system 1) processes that are not under conscious control. However, recent cognitive neuroscience research has begun to question this theory, suggesting instead that the techniques used to increase cognitive load may decrease activity in particular regions of the brain needed to make social judgments [6]. If subsequent findings were to continue to support this emerging new theory, it would change our models for conceptualizing the relationships between the cognitive processes involved with cognitive load and mental workload. As new findings are published we have the opportunity to update our models, re-interpret findings from past research, ask new research questions with a variety of research methods, and continually build our understanding of cognition.

These are only two of the many possible considerations that could be used to illustrate both the methodological and evolving theoretical challenges with which assessor cognition researchers must grapple. They also point towards important gains in the quality of assessments that may be made by more fully understanding assessors’ thinking. Regardless of the fact that the mediating influence of mental workload was not supported, Tavares and his colleagues did demonstrate that assessment judgments were enhanced by interventions based on the theory of mental workload [7, 8]. Moreover, the fact that their first intervention (i.e. reducing the number of assessment domains from 7 to 2) runs somewhat in contrast to the more prevalent strategy of encouraging comprehensive assessments by use of multiple assessment domains, means that their findings are unlikely to significantly impact practice unless they can be further evidenced and understood. As a result, Paravattil and Wilby’s review should serve to prevent this important, but incompletely understood, topic from slipping from our collective attention, and should instead stimulate the field to the need to develop it to the point where it has the impact it deserves on assessment in medical education.

It is through deep and continued explorations into surprising and paradoxical findings that we can navigate the challenges of assessor cognition research towards improved assessment outcomes. Assessor cognition research needs to take the next steps into understanding research findings and translating research in order to inform practice. Otherwise, it risks leaving the field of medical education with a series of interesting but unconnected findings that have had little impact on assessment conduct. Given the complexity of the object of study, assessor cognition researchers may do well to partner with social psychologists and cognitive neuroscientists both to support their use of methodology and understanding of theory. Paravattil and Wilby urge us to move mental workload research into real-world contexts and we agree that this is a promising direction. In order to test the mechanistic elements of our emerging understanding, however, journal editors and reviewers may want to remain open to research that uses artificial contexts to isolate and manipulate theoretically important factors. By calling our attention to the remaining inconsistencies in this topic, this article may therefore serve to assist our field in moving towards action which realises the goals which these findings promise.

## References

[1] Paravattil B, Wilby KJ (2019). Optimizing assessors’ mental workload in rater-based assessment: a critical narrative review. Perspect Med Educ.

[2] Naismith LM, Cavalcanti RB (2015). Validity of cognitive load measures in simulation-based training: a systematic review. Acad Med.

[3] Charles RL, Nixon J (2019). Measuring mental workload using physiological measures: a systematic review. Appl Ergon.

[4] Box GEP (1976). Science and statistics. J Am Stat Assoc.

[5] Bodenhausen GV, Morales JR, Weiner IB, Tennen HA, Suls MJM (2012). Social cognition and perception. Handbook of Psychology.

[6] Jenkins AC (2019). Rethinking cognitive load: A default-mode network perspective. Trends Cogn Sci.

[7] Tavares W, Eva KW (2014). Impact of rating demands on rater-based assessments of clinical competence. Educ Prim Care.

[8] Tavares W, Ginsburg S, Eva KW (2016). Selecting and simplifying: rater performance and behavior when considering multiple competencies. Teach Learn Med.

